# Effect of cupuassu butter on human skin cells

**DOI:** 10.1016/j.dib.2018.10.026

**Published:** 2018-10-12

**Authors:** Katsura Sano, Hiroko Kawanobe, Takao Someya

**Affiliations:** ALBION Co. Ltd., 1-7-10 Ginza, Chuo-ku, Tokyo 104-0061, Japan

**Keywords:** Cell number, Calcein assay, Real-time PCR, Gene expression profile, Fibroblast, Wound scratch assay

## Abstract

This study investigated the effect of cupuassu butter on the cell number of human skin fibroblasts, as well as the gene expression profiles of certain growth factors in these fibroblasts. Cupuassu butter is a triglyceride composed of saturated and unsaturated fatty acids extracted from the fruit of *Theobroma grandiflorum*. The dataset includes expression profiles for genes encoding basic fibroblast growth factor (bFGF), stem cell factor (SCF), vascular endothelial growth factor (VEGF), hepatocyte growth factor (HGF), fibroblast growth factor-7 (FGF7), and epidermal growth factor (EGF). Cell viability profile is presented as a line graph, and the expression profiles are shown as bar graphs. Furthermore, this article also describes the effects of cupuassu butter on wound healing *in vitro*. The wound healing effects are shown as a bar graph accompanied with representative microscopic images.

**Specifications table**

**Table:** 

Subject area	*Biology*
More specific subject area	*Cell biology*
Type of data	*Graph, microscope photographs*
How data was acquired	*fluorescence microplate reader, Quantitative RT-PCR, microscope*
Data format	*Analyzed*
Experimental factors	*Cell number analysis, RT-PCR analysis, Wound healing analysis*
Experimental features	*Analysis of cell number by calcein assay*
	*Analysis of gene expression by quantitative RT-PCR*
	*Analysis of wound healing effects in vitro by wound scratch assay*
Data source location	*Brazil*
Data accessibility	*Data are available within this article*

**Value of the data**

• Our data showed that changes in fibroblast cell numbers upon treatment with cupuassu butter are valuable for estimating the proliferative effect of this extract on human dermal fibroblasts.
• Cupuassu butter mediated modulation of growth factor genes of dermal fibroblasts and could be important for investigations in pharmacology and cosmetics.
• The wound healing ability of fibroblasts in response to cupuassu butter exposure provides insights for estimating the tissue repair ability of this extract.
• Our data can be used for investigations concerning the effect of cosmetics and natural medicines on human skin.

## 1 Data

**Fig. 1:**
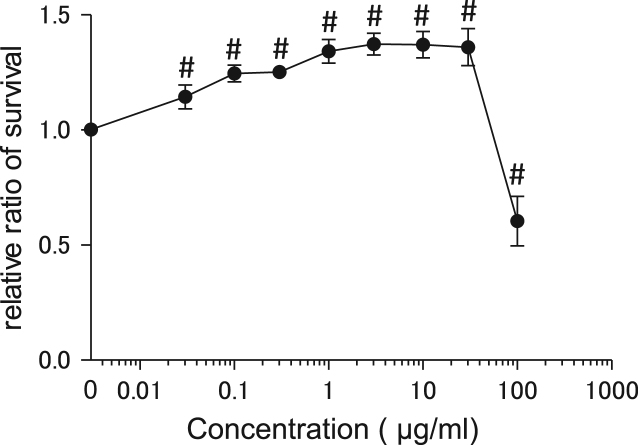
Cell viability of fibroblasts detected by calcein assay. Cells were treated with various concentrations of cupuassu butter for 24 h, and percent cell viability was calculated relative to that of untreated control. The values are shown as the mean ± SE of three independent experiments (#; *P* < 0.001 vs. control).

**Fig. 2:**
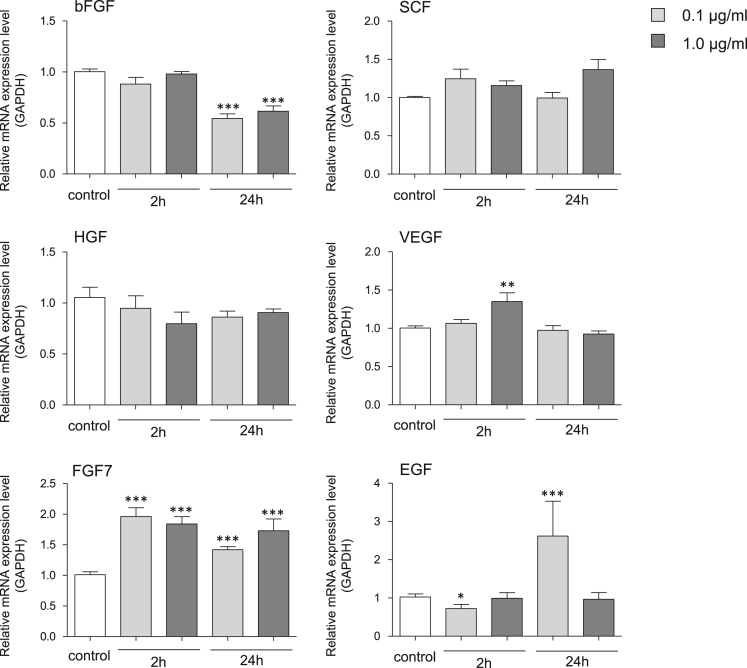
Expression levels of few fibroblast growth factors after exposure to cupuassu butter. The mRNA expression levels were normalized to GAPDH expression, and the relative gene expression levels in fibroblast cells at 2 and 24 post-treatment were compared with the corresponding levels in untreated cells, whose levels were defined as 1.0 (*P< 0.05, **P < 0.01 and ***P < 0.001 vs. control).

**Fig. 3:**
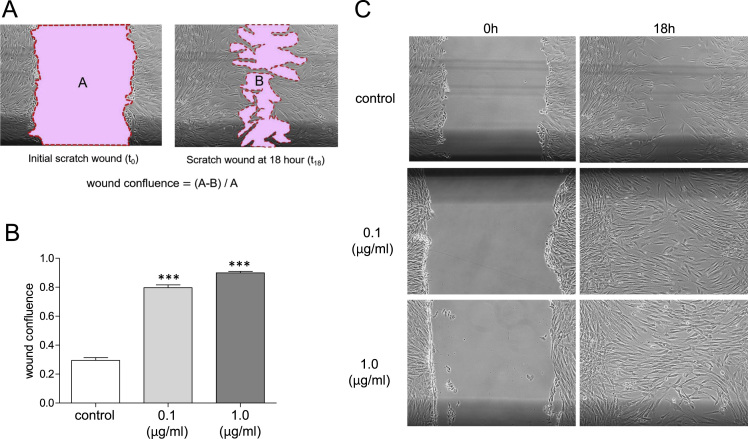
Wound healing assay using fibroblasts in presence of cupuassu butter (*P < 0.05, **P < 0.01 and ***P < 0.001 vs. control). A: Calculation of wound confluence. B: Wound confluence at 18 h post wound formation (*P < 0.05, **P < 0.01 and ***P < 0.001 vs. control). C: Digitized images at 18 h post wound formation.

First, we investigated the effects of cupuassu butter on the cell number of human skin fibroblasts. Cells were treated with various concentrations of cupuassu butter for 24 h, and cell viability percentage was calculated relative to that of the untreated control (Fig. 1). mRNA expression levels of few fibroblast growth factor genes in response to 0.1 and 1.0 μg/ml cupuassu butter treatment were then measured. The dataset included expression profiles for genes encoding bFGF, SCF, VEGF, HGF, FGF7 and EGF (Fig. 2). Finally, the wound healing ability of human skin fibroblasts in response to 0.1 and 1.0 μg/ml cupuassu butter was examined. The ratio of wound confluence was calculated relative to that of the untreated control (Fig. 3). Data are represented as mean ± SE values of triplicate independent experiments (*P < 0.05, **P < 0.01 and ***P < 0.001 vs. control).

## 2 Experimental design, materials, and methods

### 2.1 Materials

Cupuassu butter (CROPURE CUPUASSU-SO-(JP)) was purchased from Croda Japan KK. Cupuassu butter (1 mg) was dissolved in 1 ml of phosphate-buffered saline (PBS) containing 0.1% (w/v) Bovine serum albumin (BSA; fraction V, fatty acid-free) to obtain a concentration of 1 mg/ml for the stock solution, which was further diluted with culture media to obtain different final concentrations.

### 2.2 Fibroblast cell culture

Normal human skin fibroblasts, RIKEN original (NB1RGB), were provided by the RIKEN BRC through the National Bio-Resource Project of the MEXT, Japan. The cells were cultured in Minimum Essential Media-alpha (MEM-α; Life Technologies Corp.) supplemented with 10% fetal bovine serum (FBS; Biowest) and 0.2% NaHCO_3_. Cells were grown at 37 °C in a humidified incubator containing 5% CO_2_. For all experiments, human fibroblasts were seeded and incubated for 8 h with culture media containing 10% FBS. Cells were subsequently subjected to serum starvation for 16 h with serum-free MEM-α as previously described. [1], [2].

### 2.3 Cell number analysis (calcein assay)

To determine cell viability, cells were seeded (3 × 10^3^ cells/well) in a 96-well plate for 8 h with culture media containing 10% FBS. Cells were subsequently subjected to serum starvation for 16 h with serum-free MEM-α and exposed to various concentrations (0–100 μg/ml) of cupuassu butter for 24 h. The cells were then stained for 30 min at 37 °C with 10 mM calcein-AM (Dojindo) in the dark and washed with PBS. The fluorescence intensity (em/ex, 485/530 nm) of each well was measured using a SpectraMax® i3x fluorescence microplate reader (Molecular Devices). Viability was calculated as the percentage cell viability compared to that of control and are presented as the mean SE values for triplicate examines.

### 2.4 RNA isolation and quantitative real-time PCR

Cells were seeded into a 60-mm dish (5 × 10^4^ cells/dish) for 8 h with culture media containing 10% FBS. Cells were subsequently subjected to serum starvation for 16 h with serum-free MEM-α and treated with 0.1 and 1.0 μg/ml of cupuassu butter, for 24 h at 37 °C. The cells were harvested at 2 and 24 h post treatment. Total RNA was isolated from the cells using the TRI reagent (Merck) and used as a template for subsequent cDNA synthesis, using the Primescript RT reagent Kit (Takara bio Inc.). Gene specific mRNA levels were quantified using a Light Cycler 96 system (Roche) and SYBR *Premix Ex Taq* II (Takara Bio Inc.). Data were analyzed using the delta cycle threshold method, and calculated based on the Cq values, as described previously [3]. Expression of each gene was normalized to endogenous reference gene, *GAPDH*. The primers used for the qRT-PCR are shown in Table 1. All values are reported as means ± SE values of triplicate independent experiments.

**Table 1:** Nucleotide sequences of primers used in this study.

Primers	Sequences	Direction	Reference
***Quantitative real time-PCR***			
*bFGF*			
bFGF-F	3′-AGAGCGACCCTCACATCAAG-5′	Sense	[4]
bFGF-R	3′-ACTGCCCAGTTCGTTTCAGT-5′	Anti-sense	
*FGF7*			
FGF7-F	3′-CATGAACACCCGGAGCACTAC-5′	Sense	[5]
FGF7-R	3′-CACTGTGTTCGACAGAAGAGTCTTC-5′	Anti-sense	
*VEGF*			
VEGF-F	3′-GGAGAGATGAGCTTCCTACAG-5′	Sense	[6]
VEGF-R	3′-TCACCGCCTTGGCTTGTCACA-5′	Anti-sense	
*SCF*			
SCF-F	3′-GGATGGATGTTTTGCCAAGT -5′	Sense	[7]
SCF-R	3′-TCTTTGACGCACTCCACAAG -5′	Anti-sense	
*HGF*			
HGF-F	3′-AGAAATGCAGCCAGCATCAT -5′	Sense	original
HGF-R	3′-CACATGGTCCTGATCCAATC -5′	Anti-sense	
*EGF*			
EGF-F	3′-GACTT GGAGCCTG GCAGAA-5′	Sense	[8]
EGF-R	3′-CATGCACAAGTGTGACTGGAGGT-5′	Anti-sense	
*GAPDH*			
GAPDH-F	3′-GAAGGTGAAGGTCGGAGTC-5′	Sense	[9]
GAPDH-R	3′- GAAGATGGTGATGGGATTTC-5′	Anti-sense	

### 2.5 Wound scratch assay

Normal human skin fibroblasts were seeded (3 × 10^5^ cells/dish) into a 6-cm dish, and maintained at 37 °C and 5% CO_2_ until the formation of a confluent monolayer. Thereafter, cells were serum starved for 16 h with serum-free MEM-α, which was then replaced with medium containing either cupuassu butter (0.1 and 1.0 μg/ml) or the vehicle. After 24 h, the confluent monolayer of cells was scratched manually using a p200 pipette tip. The cells were washed thrice with PBS to remove scratched cells. Cell migration was monitored by collecting digitized images at 0, 6, 12, 18, and 24 h post wound formation. Digitized images were captured with an inverted microscope (Nikon) and digital camera (Nikon). These digitized images were analyzed using Image J software to measure the area of the scratched field (Fig. 3A).

### 2.6 Statistical analysis

All the values are represented as mean ± SE values. The data were analyzed using the Student׳s *t*-test. A *P* value of less than 0.05 was considered to be statistically significant.
