# Supplementary material for: Effect of cupuassu butter on human skin cells
Source: Data Brief. 2018 Oct 12;21:516–21. doi: 10.1016/j.dib.2018.10.026 (PMC6199812; doi:10.1016/j.dib.2018.10.026)
Supplement: Supplementary file 1 — Supplementary material [file mmc1.docx]

**Conflicts of interests: None**
